# The Clinical Society of Maryland

**Published:** 1892-06

**Authors:** W. T. Watson

**Affiliations:** Secretary; 1519 N. Broadway, Baltimore, Md.


### *SOCIETY REPORTS.*

### THE CLINICAL SOCIETY OF MARYLAND.

BALTIMORE, MD., April 1st, 1892.

The 25th regular meeting was called to order by the President, ROBERT W. JOHNSON.

DR. WM. S. GARDNER read a paper upon

### THE MECHANISM OF AXIS TRACTION FORCEPS.

A pair of forceps designed by the author were exhibited.

DR. FRANK DYER SANGER read a paper on

### DEATH FOLLOWING SUPRAPUBIC ASPIRATION OF THE BLADDER.

The patient was 75 years of age, white, large, rather fleshy, full habit. Had had trouble passing his urine for some time but never retention. For three days had suffered much pain in the region of the bladder and could only pass a small quantity of urine at a time. Examination showed the bladder to be moderately distended, its summit about two inches below the umbilicus. A hot bath gave no relief. A number of strictures were found in the urethra, nevertheless a long curve catheter was passed as far as the prostatic urethra; nothing could be passed further. Seven hours after the patient was first seen, aspiration was determined upon, as I felt sure the bladder would suffer if not soon relieved. A double inguinal hernia and a rather thick accumulation of fat over the pubes decided me to insert the needle well up. Having used thorough antiseptic precautions, I felt that I could pass the needle through the peritoneum with safety. About one quart of urine was removed from the bladder. A drop of blood followed the removal of the needle, the point of puncture was covered with a strip of adhesive plaster and the patient went to sleep. Next day his bowels moved freely, and he passed considerable urine, a part of which escaped into the bed and could not be measured. The morning of the second day after the operation he complained of pain in the lower part of the abdomen and tenderness. Bladder could not be felt; pulse somewhat accelerated; temperature normal. Toward evening abdomen became tympanitic, pulse more rapid, temperature 98, expression anxious, urine passed in small quantities. Bladder could not be made out. Opium given to relieve pain and heat applied to abdomen. Patient died next morning, sixty-two hours after the aspiration. *Post mortem.* Needle had entered the abdominal wall two inches above the upper border of the symphysis pubis. A line of light extravasation marked the track of the needle through the wall and parietal peritoneum fold; further than this its track could not be positively determined as the pelvic cavity was filled with blood. Dense adhesions bound the bladder in all directions which required considerable force to be broken up. There was considerable redness of the parietal and visceral peritoneum in the vicinity of the bladder. No pus or urine apparently. In freeing the adhesions about the bladder that organ was ruptured and about half a pint of turbid urine escaped. I removed the bladder and urethra *en masse* but was prevented from further examination by friends who came to claim the body. I regret that I did not at least secure one of the kidneys as it might have thrown some light on the cause of death.

There have been a number of deaths reported from supra-pubic puncture for the relief of a distended bladder. Deneffe and Van Wetter in 1877 collected 152 cases of suprapubic puncture with six deaths; eighty-seven cases of rectal puncture with eleven deaths. I have not been able to find another case of accident from aspiration in the literature, though my search has not been by any means exhaustive. Deneffe and Van Wetter report fifty-seven cases of aspiration with no accident, showing the improvement upon puncture. The case here reported proves at least that aspiration is not free from danger and suggests greater circumspectness in its practice.

DR. W. P. CHUNN: In these cases of distended bladder by sticking close to the symphysis you can get into the bladder without striking the peritoneum at all, and this is what most operators attempt to do. In this case under consideration some urine probably trickled out of the bladder and gave rise to peritonitis.

DR. J. W. CHAMBERS: I begin to look upon every case of greatly distended bladder in old men with enlarged prostate with a certain amount of apprehension. The condition is a dangerous one. The case in point is interesting from the amount of hemorrhage that followed a simple aspiration. The condition of the veins over the front of the bladder can be very aptly compared to the condition of the veins in front of the trachea where we frequently meet irregular veins which give rise to considerable trouble in operations. In the present case, with an enlarged prostate interfering with the circulation, the veins on the anterior surface of the bladder were doubtless distended and probably one of these varicose veins was punctured, giving rise to the hemorrhage. The peritoneum was probably infected by the needle which became infected in the bladder. Ordinarily a puncture two inches above the symphysis, when the bladder is distended, would not strike the peritoneum, as there is then usually two and a half to three inches space between the symphysis and the peritoneal reflection.

DR. S. K. MERRICK read a paper on

IDIOPATHIC PERICARDITIS, with report of two cases. The term idiopathic pericarditis is used by authors to define an inflammation of the pericardium (which may be acute, sub-acute or chronic) not the result of any discernible, preceding or concomitant pathological process. To eliminate every etiological factor in any given case and by exclusion arrive at a diagnosis of idiopathic pericarditis requires no little pains on the part of the practitioner. Not a few authors are skeptical as to its existence. DaCosta, while admitting its extreme rarity, says he has seen several cases about which he has no doubt as to the diagnosis. It may be that the paucity of cases of this affection reported depends in no small degree upon the obscurity of the symptoms and latency of the affection which may possibly be characteristic of this form of pericarditis.

CASE 1. Widow, aged sixty, came under observation at the

Northwestern dispensary in early part of 1887. Complained of pain in the precordial region, of great weakness and faintness on exertion and that her hands and feet were always cold. She had had no acute illness for years. Never had rheumatism nor any of the diseases which stand in an etiological relation to cardiac diseases. Her urine was examined and was found normal. Careful auscultation discovered no valvular lesions; all the valvular sounds were clearly audible, but weak. The apex beat was in the normal position but lacked force. The diagnosis made was weak heart from malnutrition, the latter being due to some unknown cause. She was slightly jaundiced, her skin being very much like parchment. She grew gradually weaker and progressively emaciated, the coldness of the extremities reaching up to the elbows and knees. To the touch she was more like a cold blooded animal than the genus homo. Her urine was repeatedly examined and was always normal. She entered the Maryland general hospital in the fall of 1887 and died about three months later. The autopsy was held by the late Dr. E. R. Walker The heart and lungs were removed, the latter being sound and free from adhesions to the pleuræ or pericardium. The valves of the heart were perfectly sound, but the walls of the heart were atrophied and thin and on close examination there was found a uniformly adherent pericardium which could be peeled off. The whole organ was firmly compressed by the adherent sac. All other organs were normal except the liver which on close examination was found to contain small points of scar tissue here and there, the sites of former localized hepatitis, no doubt. The coronary artery had doubtless been compressed by the adherent sac, and thus the nutrition of the heart had suffered.

CASE 2. Widower, aged forty-two, salesman in clothing house, admitted to the Maryland general hospital November 10, 1891. His health had been good till three weeks previous when he had considerable pain about the precordia. Said he had no fever at any time. Temperature was always normal while he was in the hospital. Urine normal. Man had never had rheumatism nor any disease to which pericarditis could be referred. No apexbeat discovered by inspection or palpation. No friction fremitus on palpation. In percussion, an increased area of dullness over lower cardiac region. Auscultation revealed the to and fro new leather sound heard with increasing loudness as the ear approached the base from the apex. A rather loud aortic regurgitant murmur was heard in the second right intercostal space, the blowing character of which was in sharp contrast to the pericardial friction sound. I pronounced it a case of pericarditis with effusion complicated with endocarditis and aortic valvular lesions. Dr. Street, who also examined the patient, came independently to the same diagnosis. The patient, a few days after coming under my care, disobeyed certain rules of the hospital and was dismissed, much to my regret.

DR. W. T. HOWARD, JR., thought that the lesions described in the liver in the first case were suggestive of syphilis. If this case could be associated with syphilis it would be most interesting, as syphilis has never been set down as a cause of pericarditis.

DR. MERRICK : I could not exclude syphilis. There were no symptoms of syphilis as long as the case was under my care. The lesions were on the surface of the liver and dipping down a quarter of an inch or so. Dr. Walker, who made the autopsy, thought they were the sites of hepatitis.

DR. HOWARD related a number of cases of adherent pericarditis which had come under his observation.

DR. A. K. BOND : I have no doubt at all that Dr. Merrick's cases were cases of idiopathic pericarditis. One cause of pericarditis and endocarditis that might sometimes be mistaken for idiopathic is rheumatism where there is no associated joint pains. In a case under my observation this winter I found signs of an old pericarditis. The patient told me that these signs had been present since childhood. She said she never had rheumatism. As in infancy and childhood rheumatism sometimes manifests itself not in joint pains but by other symptoms such as chorea, I asked the patient if she had ever had St. Vitus' Dance. She replied that she had had several very obstinate attacks.

DR. CHARLES O'DONOVAN : Quoted from Ziemsen, Loomis, Gowers and others to show the extreme infrequency of idiopathic pericarditis. In Dr. Merrick's first case a very considerable trouble was found in and about the liver, and it seems hardly proper to record this as a case of acute idiopathic pericarditis. The second case was not the subject of autopsy and is therefore incomplete. It is quite possible that some cause may have existed which was not detected. It is very plain in my mind that the first case was not one of idiopathic pericarditis.

DR. J. F. MARTENET: In ten years' special work in chest troubles I have never come in contact with a case of idiopathic pericarditis. I should think that the first case of Dr. Merrick was of syphilitic origin. I do not know that Dr. Merrick has eliminated chorea. We scarcely ever have a case of chorea persistent in character in which we do not have some trouble in the heart area. Dr. Osler says that one should look for troubles in the cardiac region associated with and following chorea. It seems to me that there must be some other affection possibly early in life, that he has not traced out.

DR. HOWARD, JR., thought it extremely improbable that a case of idiopathic pericarditis ever occurs. The second case, in which there was no autopsy he thought must be excluded.

DR. J. W. CHAMBERS: It seems to me that the interest in Dr. Merrick's case is not so much the *cause* of pericarditis but the fact that this woman died and the principal lesion was in the pericardium. Lesions of the heart, endocardium, pleuræ, kidneys and other organs, which are usually associated with pericarditis, were absent. "Idiopathic" is simply a waste-basket in which we throw things for convenience. Undoubtedly this trouble had a cause, but what the doctor means to say is that he could not find out the definite cause.

DR. O'DONOVAN: I think Dr. Chamber's remarks are hardly *apropos*. The whole world is searching for a case of idiopathic peritonitis, and as far as I know they have not been able to find it. The same holds good as to pericarditis. It is hardly the thing to claim these cases as almost isolated cases of a very rare disease. Every case should be judged on its merits.

DR. CHAMBERS: Since the whole world is looking for a thing and has not found it, it shows that it is not particularly interesting to the general practitioner. The point of interest I still think is that in that particular case the only lesion was an inflammation of the pericardium.

DR. O'DONOVAN : There was liver trouble.

DR. CHAMBERS : The marked lesion was in the pericardium.

DR. NORMENT : With Dr. Chambers and Dr. O'Donovan I doubt if there is idiopathic anything, if by "idiopathic" is meant a disease without an underlying cause. The question of associating a previous illness with the case in hand is an interesting one. Dr. Howard said that he knew of no case in which syphilis has been recorded as a cause of pericarditis. If syphilis was likely to be a cause of pericarditis in the present case, it seems to me that it would have been the cause of it in a good many other cases as well, considering the prevalence of syphilis. If a man had syphilis twenty years ago, pneumonia ten years ago, typhoid or what not five years ago, and there is not a chain connecting these diseases with the disease that takes him off, it seems hardly fair to attribute lesions that are present to-day to something that happened long since. When we cannot say what is the matter it is better to say that we do not know ; and it is in this sense that the word idiopathic is used to-day rather than in a definite sense.

DR. MERRICK : With regard to syphilis, in searching over the authorities, I could not find syphilis as ever having been the cause of pericarditis. As to rheumatism in childhood, that is readily disposed of by the character of the adhesions, which showed the trouble to be of recent date. I grant what the gentlemen have said of the extreme rarity and perhaps the impossibility of idiopathic pericarditis. All of Dr. O'Donovan's authorities acknowledge that such a thing may occur. DaCosta says there are cases in which the closest investigation has failed to show any assignable cause. In twenty years' practice, during twelve or fifteen of which I have had all the clinical material of the Northwestern Dispensary, averaging four to six thousand cases in a year, I have had an opportunity of getting hold of such a case if such a thing exists. Dr. Walker, who had made over 3,000 post mortems, particularly noted this case in the hospital and that was the reason an autopsy was held.

The case was a unique one in Dr. Walker's experience.

DR. W. T. WATSON, *Secretary.*

1519 *N. Broadway, Baltimore, Md.*

